# Radiologist-like artificial intelligence for grade group prediction of radical prostatectomy for reducing upgrading and downgrading from biopsy

**DOI:** 10.7150/thno.48706

**Published:** 2020-09-02

**Authors:** Lizhi Shao, Ye Yan, Zhenyu Liu, Xiongjun Ye, Haizhui Xia, Xuehua Zhu, Yuting Zhang, Zhiying Zhang, Huiying Chen, Wei He, Cheng Liu, Min Lu, Yi Huang, Lulin Ma, Kai Sun, Xuezhi Zhou, Guanyu Yang, Jian Lu, Jie Tian

**Affiliations:** 1 School of Computer Science and Engineering, Southeast University, Nanjing, China.; 2 CAS Key Laboratory of Molecular Imaging, Beijing Key Laboratory of Molecular Imaging, the State Key Laboratory of Management and Control for Complex Systems, Institute of Automation, Chinese Academy of Sciences, Beijing, China.; 3 Department of Urology, Peking University Third Hospital, Beijing, China.; 4 Urology and lithotripsy center, Peking University People's Hospital, Beijing, China.; 5 Department of Radiology, Peking University Third Hospital, Beijing, China.; 6 Department of Pathology, Peking University Third Hospital, Beijing, China.; 7 LIST, Key Laboratory of Computer Network and Information Integration, Southeast University, Ministry of Education, Nanjing, China.; 8 Engineering Research Center of Molecular and Neuro Imaging of Ministry of Education, School of Life Science and Technology, Xidian University, Xi'an, China.; 9 CAS Center for Excellence in Brain Science and Intelligence Technology, Institute of Automation, Chinese Academy of Sciences, Beijing, China.; 10 Beijing Advanced Innovation Center for Big Data-Based Precision Medicine, School of Medicine and Engineering, Beihang University, Beijing, China.; 11 Key Laboratory of Big Data-Based Precision Medicine (Beihang University),Ministry of Industry and Information Technology, Beijing, China.; 12 School of Artificial Intelligence, University of Chinese Academy of Sciences, Beijing, 100080, China.

**Keywords:** prostate cancer, Gleason grade groups, deep reinforcement learning, prostate cancer grading, magnetic resonance imaging

## Abstract

**Rationale:** To reduce upgrading and downgrading between needle biopsy (NB) and radical prostatectomy (RP) by predicting patient-level Gleason grade groups (GGs) of RP to avoid over- and under-treatment.

**Methods:** In this study, we retrospectively enrolled 575 patients from two medical institutions. All patients received prebiopsy magnetic resonance (MR) examinations, and pathological evaluations of NB and RP were available. A total of 12,708 slices of original male pelvic MR images (T2-weighted sequences with fat suppression, T2WI-FS) containing 5405 slices of prostate tissue, and 2,753 tumor annotations (only T2WI-FS were annotated using RP pathological sections as ground truth) were analyzed for the prediction of patient-level RP GGs. We present a prostate cancer (PCa) framework, PCa-GGNet, that mimics radiologist behavior based on deep reinforcement learning (DRL). We developed and validated it using a multi-center format.

**Results:** Accuracy (ACC) of our model outweighed NB results (0.815 [95% confidence interval (CI): 0.773-0.857] vs. 0.437 [95% CI: 0.335-0.539]). The PCa-GGNet scored higher (kappa value: 0.761) than NB (kappa value: 0.289). Our model significantly reduced the upgrading rate by 27.9% (*P* < 0.001) and downgrading rate by 6.4% (*P* = 0.029).

**Conclusions:** DRL using MRI can be applied to the prediction of patient-level RP GGs to reduce upgrading and downgrading from biopsy, potentially improving the clinical benefits of prostate cancer oncologic controls.

## Introduction

Originally introduced in the 1960s, the Gleason score (GS) remains the most powerful prognostic factor for prostate cancer (PCa) 1. In 2014, a new five-category GS grade group (GG) system was established. Gleason categories having similar prognoses are grouped into certain GGs: GS 3 + 3 = 6 is GG 1; GS 3 + 4 = 7 is GG 2; GS 4 + 3 = 7 is GG 3; GS 4 + 4 = 8 is GG 4, and GS 9-10 is GG 5 2. This system has been widely adopted and was recently validated 3-5.

However, PCa was diagnosed mainly by applying systematic needle biopsies (NB), which has limited prognostic power. Accurate identification of GGs from the biopsy was essential to risk stratification and decision-making in clinical practice. Discrepancies between the GS of NB and radical prostatectomy (RP) pathology have been reported worldwide. The upgrading rates of NB to RP range from 26% to 36% and downgrading rates range from 5% to 14.7% 6-8. Such changes have led to overtreatment or undertreatment for patients.

Magnetic resonance imaging (MRI) has been adopted for PCa diagnosis and staging. MRI can overcome bias and insufficiency by sampling in a non-invasive manner to elucidate the inherent nature of tumoral heterogeneity 6-8. Several studies have shown the efficacies of using MRI to predict GS/GG 10,11. The Prostate Imaging Reporting and Data System (PI-RADS V2.1) has demonstrated potential for GS/GG prediction, but sensitivity and specificity remained at approximately 70%, according to previous studies 12-14. Moreover, several studies correlated texture and shape features based on radiomics 15 were developed for GS/GG predication. The area under curves (AUCs) of these studies were from 0.64 to 0.83 16,17. Therefore, there is an acknowledged need to improve the accuracy to a clinical-grade level in future iterations of methods.

Deep learning and big data 18 were powerful tools for further increasing the diagnostic quality of GS/GG independent of human annotations. Studies that have implemented deep learning algorithms to predict GS/GG in prostatectomy specimens, biopsies, and microarrays have been recently reported 19,20. In these, attention was paid primarily to GS-7 cases at biopsy core specimens. Unfortunately, few studies have attempted to build a patient-level hierarchical prediction model to support clinical decision-making by addressing up-/downgrading alterations 21. Notably, deep reinforcement learning (DRL) has an advantage with complex reasoning tasks because of its environmental perception, which could provide an analysis approach from slice-level results to patient-level results 22. DRL has shown its potential in lesion detection, diagnosis, and segmentation 23. However, it has not been effectively applied to grade prediction using MRI.

In this study, we developed and validated an interpretable framework that mimicked human-behaviors to decrease the risk of up-/downgrading from NB to RP pathologies. Our framework predicted the final GG at the “patient-level” without manual annotations. Additionally, with the aid of our algorithm, clinical benefits based on predicted GG results were evaluated and validated against multi-center datasets.

## Materials & Methods

### Patients

**Figure 1:**
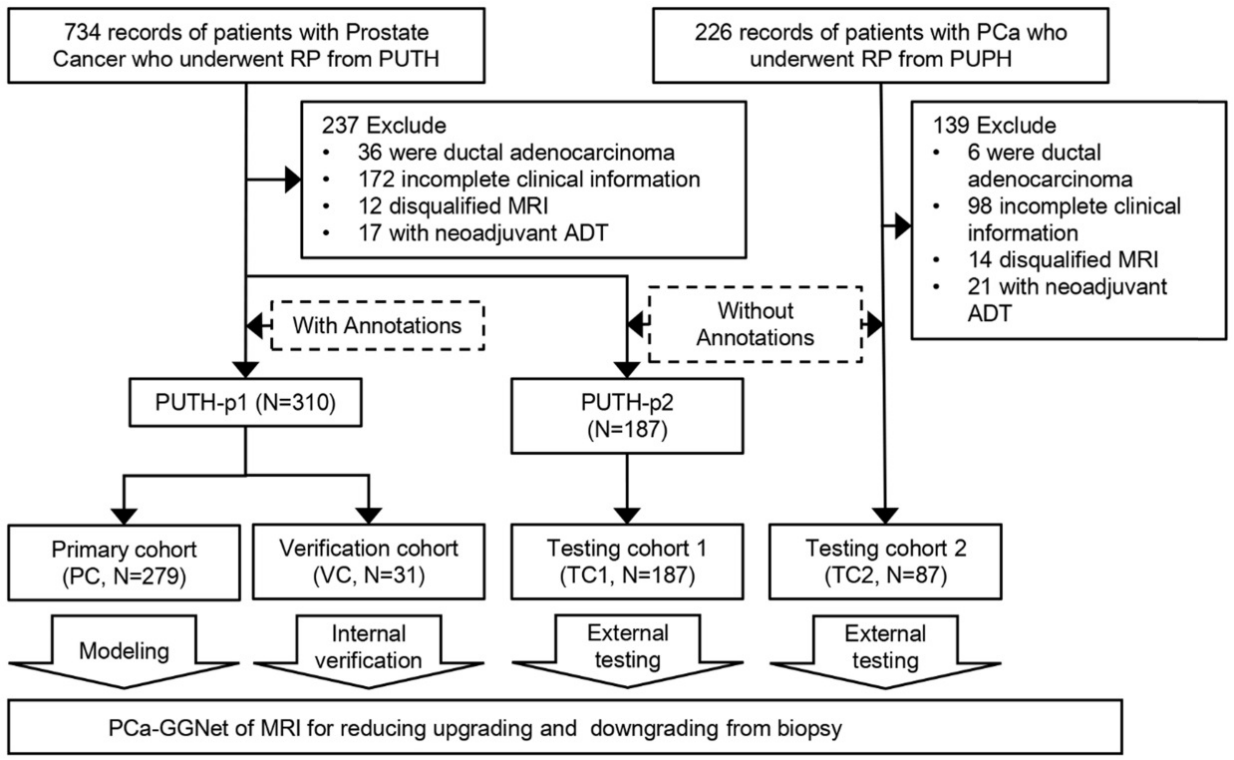
Patient recruitment and study design.

From the hospital information system of Peking University Third Hospital (PUTH) and Peking University People's Hospital (PUPH), we enrolled patients who underwent RP between January 1, 2010 and December 31, 2019. All patients underwent ultrasonography-guided transrectal systematic biopsy (SBx) with a maximum of 14 cores at each hospital. Patient-level GS of needle biopsies and prostatectomies were documented and converted to GG form. All patients received magnetic resonance (MR) exams 1-7 days before the biopsy. We performed systematic biopsy in a transrectal manner using ultrasonography guidance, which is likely known to cause prostate-rectus adhesion. To avoid rectal injury, we opted to perform the RP at least one month after the biopsy. Our inclusion criteria were as follows: 1) prebiopsy 3T MRI of T2-weighted image with fat suppression (T2WI-FS); 2) complete documentation of clinical parameters, including total prostate-specific antigen (PSA), clinical T stage, biopsy core-level documentation, pathological tumor node metastasis staging system and relevant perioperative parameters; and 3) pathologically confirmed prostate adenocarcinoma. 4) pathological specimens of needle biopsy and RP. The exclusion criteria were as follows: 1) incomplete clinical information (the lack of GG or pathological specimen); 2) presence of other pathological types; 3) presence of distal metastasis; and d) neoadjuvant androgen deprivation therapy (ADT) cases **(Figure 1)**.

**Figure 2:**
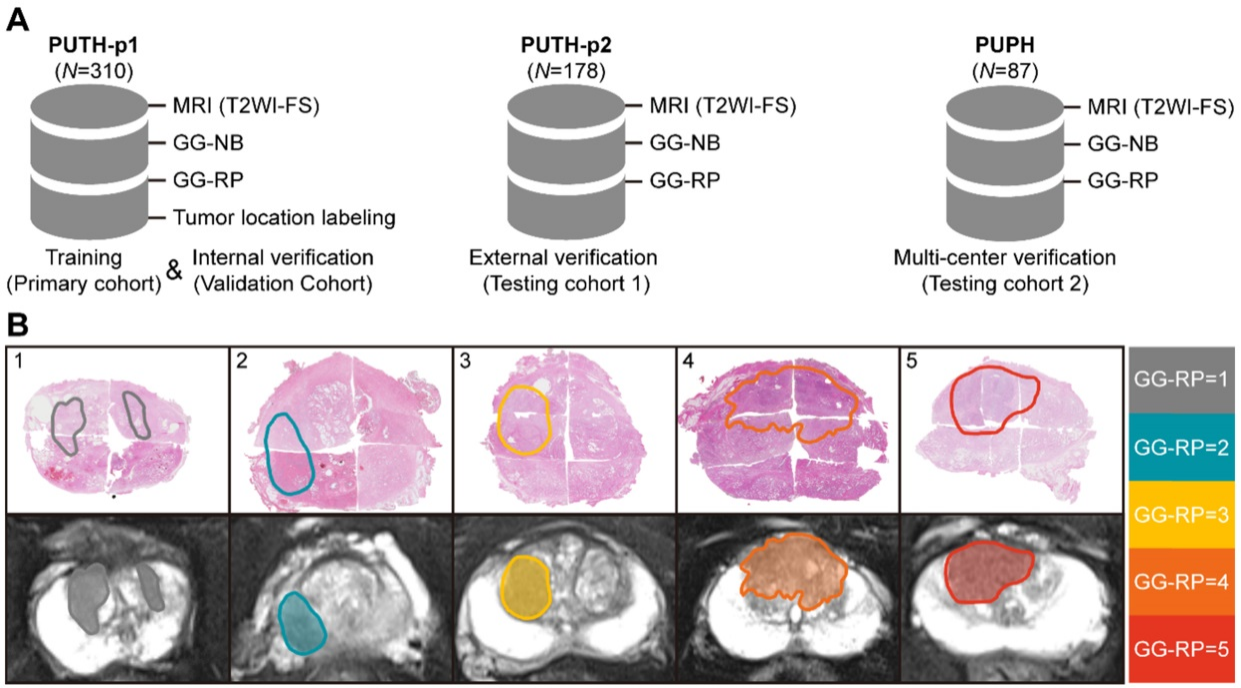
Dataset and annotations. **(A)** Multi-center datasets. **(B)** Tumor segmentation based on whole slide images of hematoxylin-eosin staining sections from different cases of RP.

We included 575 patients with pretreatment MR images, biopsy pathological results, and pathological results of RP from two Chinese hospitals. Patient characteristics are listed in **Table 1**. Patients were divided into a primary cohort (PC, *N* = 279, from PUTH-p1), internal verification cohort (VC, *N* = 31, from PUTH-p1), external testing cohort 1 (TC1, *N* = 178, from PUTH-p2) and external testing cohort 2 (TC2, *N* = 87, from PUPH) (**Table S2**). PC was used for model training. VC was used for internal verification. TC1 and TC2 were used for multi-center validation (**Figure 1, Figure 2A**).

### Imaging data acquisition and annotations based on computational pathology registration

All MRIs were performed before SBx using 3T MR scanners (Magnetom Trio, Siemens Healthcare, Erlangen, Germany/Discovery MR750, GE Healthcare, USA) without an endorectal coil. Only DICOM data of T2WI-FS (turbo-spin echo or fast-recovery fast-spin echo with fat suppression) were used for analysis in this study **(Table S1)**.

Pathological hematoxylin-eosin sections of each patient from RP were scanned at 40× magnification to computational pathological sections (NanoZoomer S360, HAMAMATSU, Hamamatsu City, Japan). First, a pathologist having 22 years of urology expertise patched all the pieces into whole-mount sections and delineated the lesions that were responsible for diagnosis on each section. Second, our pathologist and one urological radiologist (12 years of experience) together recognized and delineated lesions on MRI correlated to whole-mount images, by using the knowledge of shape, texture, location of both the prostate and the tumors, which is knowing as cognitive registration. Of note, only lesions responsible for patient-level GG assessment were delineated. Very small satellite lesions or lesions contribute little to the final diagnosis were ignored. Five different examples are shown in **Figure 2B** (**Supplementary Information I**).

In our study, the T2WI-FS contained 24 18-24 (Median [Min-Max]) slices per patient, in which 9 8-12 (Median [Min-Max]) slices containing prostate gland were included. The number of annotations per case was 5 4-10 (Median [Min-Max]). There was no significant difference between datasets in the distribution of the five-category GG-RP (*P* > 0.05).

### GSs/GGs of NB and RP

All patients received transrectal systematic biopsy at both centers. No targeted biopsy was performed. The number of cores ranged from 12 to 14. Subsequently, laparoscopic RP was performed one month after biopsy at either PUTH or PUPH. GS/GGs of NB and RP were reported at core, specimen, and patient levels according to the 2016 World Health Organization five-tier criteria 2. Each pathology report was read and verified by two board-certified pathologists having PCa experiences of 6 and 22 years, independently. For cases having different assessments, a thorough discussion was conducted to reach a final agreement (**Figure S1**).

### Deep CNN for slice-level analysis using identified tumor slices

**Figure 3:**
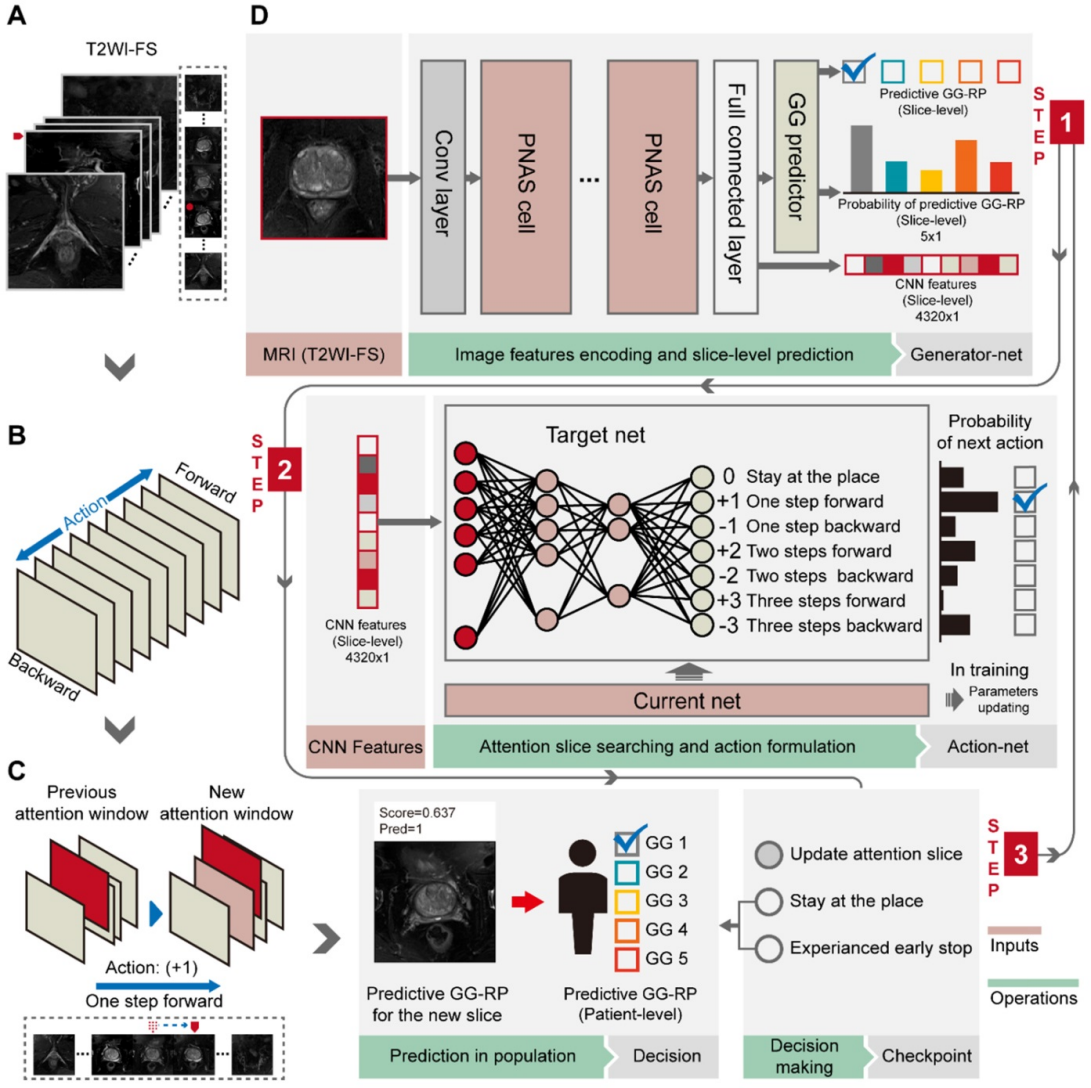
Workflow of PCa-GGNet. **(A)** The input of PCa-GGNet, for which only one slice was input per operation. The initial input was the median slice of the whole T2WI-FS sequence. **(B)** Action rules for attentional slice searching, which included direction and step length of actions. **(C)** The illustration of attentional slice searching and updating. **(D)** The workflow and architecture of PCa-GGNet. PNAS refers to a progressive neural architecture search. In the first step, we selected the median slice of T2WI-FS as input for the convolutional neural network (CNN)-based model to predict GG-RP on each slice. For the second step, we used features from the first step of the DRL-based model to generate an action for updating input. For the third step, a checkpoint was used to determine whether the results of the current input could be used as patient-level predictions. If so, the input was a decision slice. If not, our attention was changed through actions to find a new slice as an input. The framework was a sequential method to predict patient-level GG-RP. The gray arrow shows a forecasting process. See Methods for complete details.

First, we performed a pixel-wise analysis to obtain slice-level prediction and CNN features using tumor slices of T2WI-FS (**Figure 3A, 3D**). A PNASNet-5-large 24-based progressive search strategy was adopted as the structure for constructing a classification model (generator-net), which earned state-of-art performance for image classification with an accuracy of 1,000-category on the ImageNet 25 test set: 82.9% (top-1) and 96.2% (top-5). Model parameters of the model trained by ImageNet were used for the pre-training network and for transfer learning 26, in which the filter parameters of the network were frozen, except for the last five layers. Next, the model was trained with semi-supervised learning, and the label of each slice was consistent with patient-level GGs. During model training, data augmentation was used to restrict overfitting, including random rotation, mirror transformation, and affine transformation. The central point of the original image window was the anchor point and the area with a window size of 200 × 200 (pixels × pixels) near the anchor point was selected as the region of interest (ROI) to focus the network's attention on the prostate area. The ROI was then scaled to 331 × 331 (pixels × pixels) via bilinear interpolation as input.

Additionally, inputs were converted into a three-channel image, and each channel was standardized with a mean of 0.5 and a variance of 0.5. CNN features related to PCa GGs were then extracted from the last fully connected layers, providing a vector (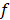) of 4,320 × 1. The output of the classifier was generated by softmax, having a vector (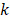) of 5 × 1, including the corresponding prediction probability of the five-grade GG, 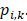 The category having maximum prediction probability contained the predicted GGs. During training, the batch size of every interaction was 64, and the loss function was defined by the cross-entropy of multiple classifications to update filters via backpropagation **(F-1)**. The consistencies between labels and predicted results were binary (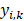). The learning rate was set to 0.01 with an exponential reduction of 0.97, and the momentum was adjusted at 0.9. When the epoch training finished, the VC was employed for internal validation and early stopping to prevent overfitting. The model training was terminated and saved until the overall ACC of five consecutive epochs was stagnant, giving us the generator net.


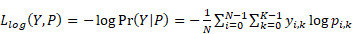
**(F-1)**

### DRL for simulating radiologist reading behavior to search for attentional slices

We used a DRL strategy to mimic the diagnostic behavior of radiologists and to improve slice-level-to-patient-level prediction. The model slid each slice of the 3D T2WI-FS forward or backward using an attention mechanism. It then associated the memory of the browsing path to empower the attentional slice searching strategy. A slice was finally obtained as the decision slice, identifying the most critical slice for the patient-level GG-RP. For the experiments, we adopted the deep-Q network (DQN) 22, which has been used to replicate the human-level player performance in sports video games, as the basic structure. We redesigned the game mechanism and reward function for the GG prediction problem. The DQN consists of current and target nets having the same configuration as an artificial neural network with two hidden layers. The input of net *(s)* is a 4,320 × 1 vector (denoted as the status), and the output *(Q)* is a 7 × 1 vector that indicates different orders of action (a) (**Figure 3B, 3D**). The two hidden layers were constructed with 50 and 30 neurons, respectively. The current net was used to collect experiences into a pool during training and to update their parameters using *Q_loss* **(F-2).** The collected experience included the rewards, *r*, underlying the current status, and actions. Rewards are defined by their predicted probability, *P_s,a_*, and the consistency between the predicted and true labels **(F-3)**. The basic reward (*y_s,a,_*) and the reward rate (α) of predicted probability (*P_s,a_*) were set as 1 and 0.5. When the experience pool overflowed, the benefits of a single action in the experience pool were randomly recorded, and the neuron parameters of the current net were assigned to the target net when the number of accumulations reached 100. The training environment included patients who provided 3D T2WI-FS slice imagery with identified CNN features. We changed the training environment by randomly selecting the starting slice to achieve data augmentation, which increased the robustness of the model. During the attentional slice searching phase, only the target net was used as a decision agent to determine the probability of actions and to select the action having the highest probability.


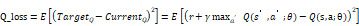
**(F-2)**


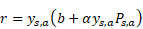
**(F-3)**

### Patient-level prediction of PCa-GGNet framework

To construct a GG prediction indicator at the patient-level, a three-stage PCa-GGNet framework was developed. Two basic units (i.e., generator and action net) were prepared during the training phase. A slice was the framework's input. A classifier based on the tumor slice was established for five-category prediction at the slice-level (i.e., generator net) (**Figure 3D**). Inputs for the training generator net included tumor slices, and their labels reflected the patient-level GG-RP. Next, the action net was trained for attentional slice searching using features and classification results, as generated by the generator net. To train the action net, we defined the T2WI-FS slices as the environment, for which labels included patient-level GG-RP and flags of tumor slices. The generator net and action net were built step-by-step. During the prediction phase, three steps were required for the PCa-GGNet framework to predict patient-level GG. In this first step, the middle slice was selected as the input to the generator net (**Figure 3A**), and CNN features and predictions based on slices were generated from the generator net. During the second step, CNN features were employed for the action net to produce an action order based on rules (**Figure 3B**). Lastly, a checkpoint was set to draw a conclusion based on the action order from the action net. If the framework-running circle was not satisfied with the condition of the checkpoint, which would experience an early stop or stay-in-place action, the framework would update the current attentional slice and repeat steps 1 and 2 (**Figure 3C**). Otherwise, the patient-level prediction would adopt the result of the attentional slice generated from the last circle. The initial input was the median slices, which recorded the radiological information of the prostate area.

### Evaluation

Quantitative statistics were summarized as mean ± standard deviation. Categorical variables were achieved via the χ^2^ test or Fisher's test. The reported statistical significance levels were all two-sided, with the statistical significance level set to 0.05. ACC and quadratic Cohen's kappa coefficient were used to evaluate the overall performance of the multi-category classification. Precision **(F-5)**, recall **(F-6)**, and F1-score **(F-7)** were used for evaluation within the category. The 95% confidence interval (CI) values were calculated using a bootstrap strategy (*N* = 1,000). Statistical analyses were performed using Python's (v.3.6.5) scikit-learn package (v.0.21.3) and R (v.3.1.0).


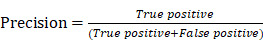
**(F-5)**


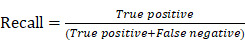
**(F-6)**


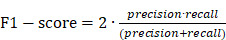
**(F-7)**

## Results

### Inconsistency between biopsy assessment and RP pathology

**Figure 4:**
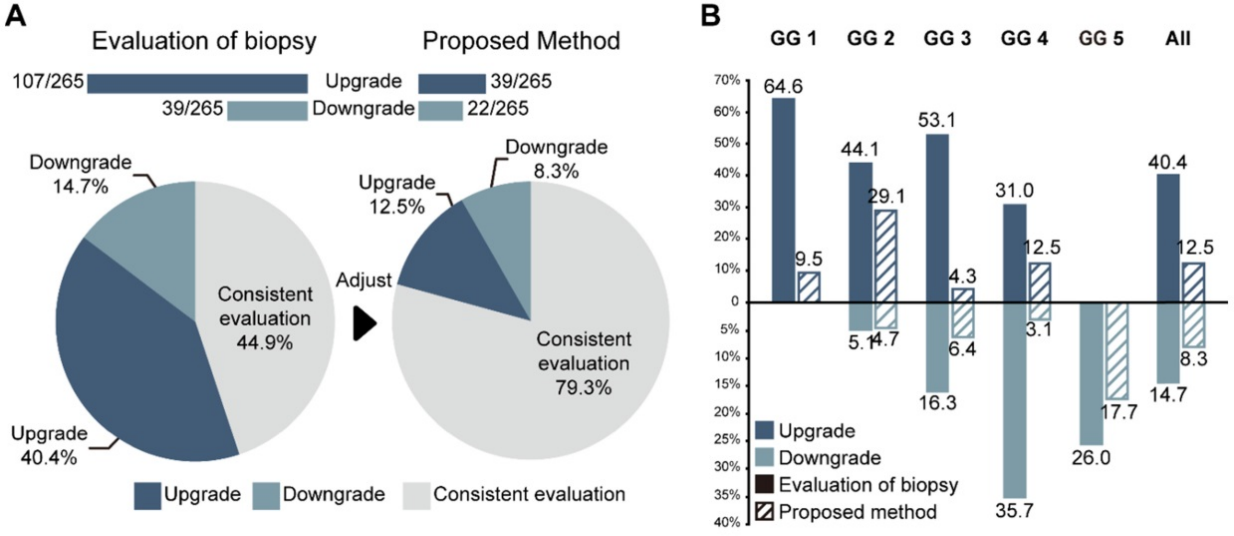
Performance of PCa-GGNet against GG-NB for upgrading or downgrading evaluation. **(A)** Overall performance. The bar chart and ratio at the top indicate the number of people upgraded or downgraded in the dataset. **(B)** Comparison of the rates of upgrading or downgrading between biopsy and our method.

**Figure 5:**
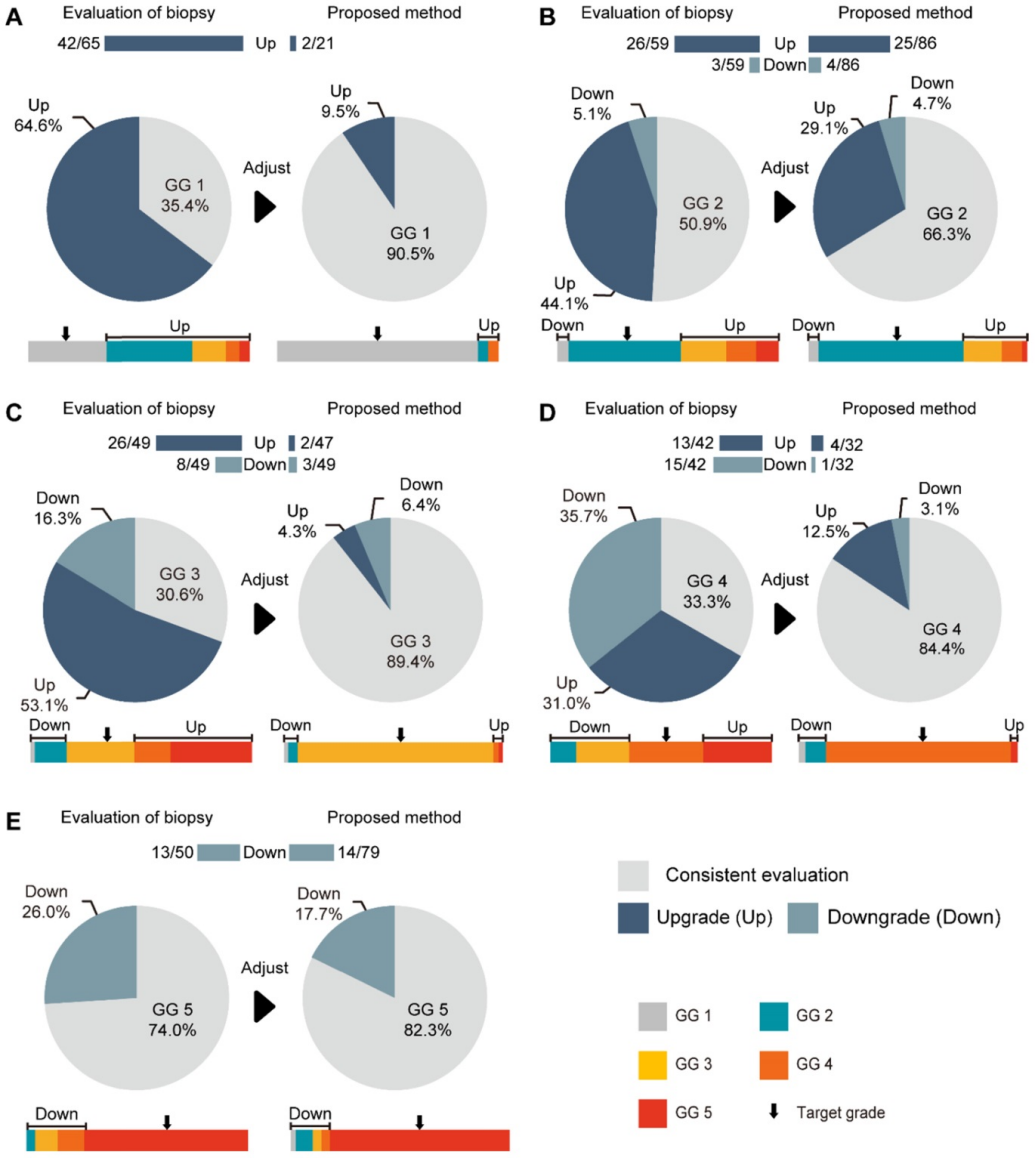
Comparison between our method and GG-NB at each grade. **(A)** Grade 1 of GG-RP. **(B)** Grade 2 of GG-RP. **(C)** Grade 3 of GG-RP. **(D)** Grade 4 of GG-RP. **(E)** Grade 5 of GG-RP. The upper bar chart and ratio at the top indicate the number of people who were upgraded or downgraded in the dataset. The color bar represents the detailed type of upgrading and downgrading group. The black arrow indicates the target of the GG-RP corresponding to the pie chart.

The consistencies between the Cohen's kappa values of GG-NB and GG-RP were 0.364 and 0.289 at PUTH and PUPH, respectively. The mean accuracy (ACC) between GG-NB and GG-RP on the total patients was 0.484 (95% CI: 0.379-0.588). The overall upgrading rate reached 40.4%, which was significantly higher than the downgrading rate of 14.7% (*P* < 0.001) (**Figure 4**). The upgrading and downgrading of each GG-NB are shown on the left side of **Figure 5.** Apart from GG 1, the second-largest proportion of upgrading was in GG 3, and the largest downgrading cases were in GG 4. Importantly, more than 50% of GG-NB 3 upgrades shifted to GG-RP 5, and some patients in GG-NB 4 or 5 downgraded to 3 or lower. Consistency analysis of GGs between GG-NB and GG-RP is shown in **Table S3** for different cohorts.

### Assessment of generator net for slice-level GG-RP prediction based on lesion slice analysis

To construct a multi-classification model for predicting patient-level GG-RP, we first built a five-category prediction model (i.e., generator net) based on lesion-level analysis to distinguish different GG-RPs as accurately as possible. During the training phase, a total of 1,484 T2WI-FS tumor slices in PC were used for the model's parametric learning. A total of 160 tumor slices from VC were used as internal verification and were regarded as the model's early terminating conditions to prevent overfitting.

The ACC of generator net for the five-category (GG-RP 1-5) classification in PC and VC were 0.73 (95% CI: 0.711-0.749) and 0.615 (95% CI: 0.545-0.686), respectively (**Table S4**). More details (i.e., precision, recall, and F1-score) at each grade are listed in **Table S5.** We also tried to merge slices without tumor annotations into original samples as a separate category to construct a six-category model to not only distinguish five levels of GG-RP, but also to filter-out slices without tumors. Although the overall ACC of the classifier was improved to 0.838 (95% CI: 0.83-0.847) in PC and 0.803 (95% CI: 0.777-0.829) in VC, the ACC for GG-RP prediction (slice-level) significantly dropped to 0.54 in PC (95% CI: 0.517-0.562) and 0.523 (95% CI: 0.451-0.594) in VC (**Table S4**), respectively. Therefore, the five-category model containing tumor annotations of training samples was adopted as the generator net for predicting GG-RP at the slice-level. We also compared the classification performance of different network structures in **Table S4**, and the most optimal basic network structure was the PNASNet-5-large net.

### Performance of discriminator net for attentional slice searching

Based on features from the generator net output, the action net was modeled for attentional slice searching to update the input of the generator net in a new prediction period and to draw the final decision. The ACC of the action net (designed to identify slices containing tumors in the 3D T2WI-FS) was 0.862 (95% CI: 0.85-0.874) in PC by five-fold cross-validation **(Table S6).** According to the rules, no matter whether if we received a “stay at the place” status at the last step, we adopted the last-searched slice as the attentional slice, so that the model would keep the sensitivity of 100%. The specificity of the model was 0.86 (95% CI: 0.848-0.872) on PC. In the experimental attempts, the four-circle was set as the terminating condition of the action net, in which the slice at the fourth act was used as the basis for the final decision. Next, we verified the action net in VC with an ACC of 0.797 (95% CI: 0.754-0.841) and a specificity of 0.797 (95% CI: 0.754-0.841) (**Table S6**). The ACC of the GG-slice to the finally selected slices was 0.86 (95% CI: 0.846-0.874) in PC and 0.832 (95% CI: 0.784-0.88) in VC (**Table S6**).

### Assessment of PCa-GGNet for predicting GG-RP at patient-level and restriction of upgrading and downgrading risks

**Figure 6:**
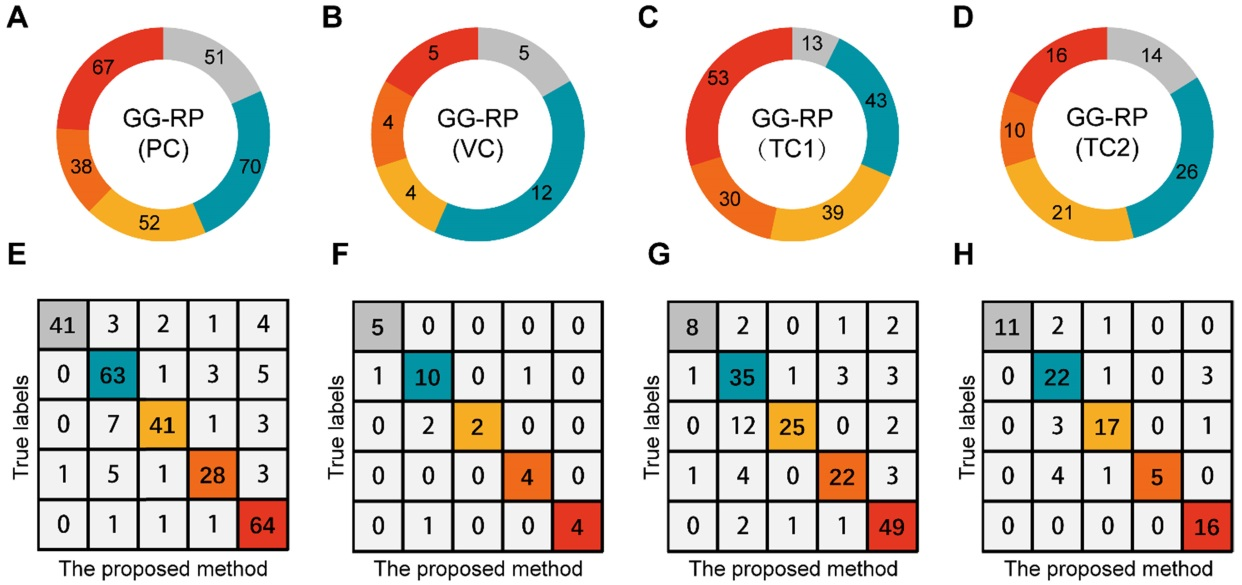
Assessment of PCa-GGNet in multi-center for multi-category prediction**. (A-D)** Proportion distribution of five levels of GG-RP in the primary cohort (PC), validation cohort (VC), testing cohort 1 (TC1), and testing cohort 2 (TC2). **(E-H)** Confusion matrix.

To explore whether the PCa-GGNet using T2WI could construct a prediction index highly related to the GG-RP at the patient-level, we first constructed a computing framework and trained it in PC. The prediction process is visualized in **Figure S2.** The predicted GG from PCa-GGNet (GG-Pre) obtained a five-category ACC of 0.847 (95% CI: 0.826-0.867) in PC and 0.83 (95% CI: 0.762-0.898) in VC (**Table S3**). The kappa consistency between the GG-Pre and GG-RP was 0.804 (95% CI: 0.752-0.857) and 0.777 (95% CI: 0.599-0.954) in PC and VC, respectively. The F1-score of each GG (1-5) was 0.893 (95% CI: 0.767-1.018), 0.79 (95% CI: 0.697-0.883), 0.62 (95% CI: 0.341-0.899), 0.868 (95% CI: 0.725-1.012), and 0.877 (95% CI: 0.732-1.022), respectively (**Table S7**). Furthermore, to validate the reliability of the PCa-GGNet, the model was tested on multi-center datasets obtaining ACCs of 0.781 (95% CI: 0.751-0.811) in TC1, and 0.815 (95% CI: 0.773-0.857) in TC2 (**Table S3**). The kappa consistency in TC1 was 0.713 (95% CI: 0.632-0.794) and 0.761 (95% CI: 0.656-0.865) and TC2, respectively. The confusion matrix between GG-Pre and GG-RP under multi-center settings are shown in** Figure 6E-H**. ROC analysis was used for evaluating the performance of GG-Pre according to different subgroups, AUCs of low-grade (grade 1 vs. 2,3,4,5), medium-grade (grade 1,2 vs. 3,4,5) and high-grade (grade 1,2,3 vs. 4,5) groups were all greater than 0.8 in PC, TC1, and TC2 (**Figure S3**).

For assessing the restriction of upgrading and downgrading risks, the inconsistency of PCa-GGNet in all testing samples decreased to 12.5% (upgrading) and 6.3% (downgrading) (**Figure 4A**). Consistency ratios of PCa-GGNet at each GG (1-5) were 90.5%, 66.3%, 89.4%, 84.4%, and 82.3%, respectively (**Figure 5**). Top-2 predictions of PCa-GGNet were in grade 1 with an F1-score of 0.876 (95% CI: 0.805-0.947) and in grade 5 with an F1-score of 0.884 (95% CI: 0.826-0.942) (**Table S7**).

Compared with GG-NB, GG-Pre reduced the overall upgrading rate by 27.9% (*P* < 0.001) and reduced the overall downgrading rate by 6.4% (*P* = 0.029). The risks of upgrading and downgrading were diminished (**Figure 4B**). The consistency in GG 1 increased to 90.5% by applying our method (*P* < 0.001). Furthermore, the proportion of GG 1 shifting to GG 5 was eliminated (**Figure 5A**). Compared with GG-NB, the upgrading rate in GG 2 decreased by 15% in our prediction (*P* = 0.093). Moreover, shifts from GG 2 to 4 or 5 were significantly reduced. In GG 3, the upgrading rate dropped from 53.1% to 4.3% (*P* < 0.001), and the downgrading rate decreased from 16.3% to 6.4% (*P* = 0.201) (**Figure 5C**). The up-/downgrading rates of GG 4 were reduced by 18.5% (*P* = 0.112) and 32.6% (*P* = 0.002), respectively, and the proportion of shifting from GG 4 to 5 decreased by one third (*P* = 0.012) (**Figure 5D**). Among all cases, the rates of GG raising two or more levels were reduced from 18.5% to 4.5%. Shifts from below GG 3 to 4 or 5 were reduced by 48.1%, among which the ratio of GG 1 shifting to 5 was eliminated. Among all grades, GG 3 was the group that obtained the highest cumulative gains for both upgrading and downgrading improvements.

## Discussion and Conclusion

For the past six decades, GS has remained one of the most powerful predictive factors for biochemical relapse and overall survival of PCa. Current treatment options are mainly decided via risk stratifications or nomograms, which consist of total PSA level, clinical T stage, NB Gleason GGs, and other clinicopathological parameters. Thus, precise assignments of biopsy GGs are crucial when making optimal treatment choices for patients. However, discrepancies between NB and RP pathology are common, and the latter is considered to reflect more accurate information about the nature of the tumor. Upgrading from NB to RP was reported to be as high as 36% 27, whereas downgrading was reported to have a lower average of 5% 1,8. Tumor aggressiveness was usually underestimated in NB-upgraded cases, followed by worse prognoses of biochemical-free survival. Corcoran et al. reported that 28.6% of upgrading cases correlated with a higher risk of biochemical recurrence 28. Boorjian et al. constructed a multivariate model to predict biochemical recurrence following RP in a cohort of over 8,000 patients, and the NB results demonstrated minimal additional value as compared with RP Gleason results 29. Similar situations were observed in a Korean population cohort, in which upgraded cases demonstrated worse biochemical-free survival and worse metastasis-free survival 30.

The reasons for discordance between NB and RP are variable, such as tumor heterogeneity, sampling bias on needle biopsies, erroneous interpretation on inadequate tissue, and different practices of GG assignments at the core- or patient-levels. To achieve more accurate results of NB pathology, numerous attempts have been made. Several studies have tried to incorporate multiple clinical parameters (e.g., PSA, core length, percentage of Gleason pattern 4) to develop models or nomograms to predict final RP results. However, the robustness and discriminative power of these models remained below the desired threshold of 0.70 31-33. This situation was improved with the adoption of MRI in targeted biopsies (TBx). Level-1 evidence from the PRECISION trial demonstrated that MR fusion TBx improved the detection rate of clinically significant PCa 34. TBx can reduce upgrading and improve tumoral percentage at each core, compared with SBx 35-38. Additionally, there is an increasing trend regarding the application of data science for automated GG scoring. Several automated deep learning algorithms for GG have been proposed on biopsy histology or tissue microarrays, producing accuracy ranges from 80% to 98% 39-41. However, most of these studies focused on biopsy samples, and limited works have addressed upgrading NBs. Furthermore, to the extent of our knowledge, few works have been accomplished for predicting the final GG from an MRI to elucidate better discrepancies between NB and RP pathology 42-45.

The black-box feature of deep learning is often regarded as the main drawback of these artificial systems, especially for treatment-related decision-making in clinical practice. The method of voxel analysis has generally not been recommended for modeling the prostate MRI because of the weakened anatomical consistency caused by the distance between imaging layers and computational costs. Overwhelmed by redundant information, pixel-wise analysis of slices has also been a big challenge, owing to the naive statistics of slice-level results. To make our algorithm more interpretable and accurate, we proposed a radiologist-like computing framework for MRI for end-to-end prediction of GG-RP, named PCa-GGNet. This tool combined the dual advantages of the pixel-wise analysis of deep learning 46 and the dynamic programming of DRL 47. In the current design, we defined each patient's T2WI-FS as a game, each imaging layer as a frame and each action as a gamer's movement. The current framework was only designed for a single sequence (T2WI-FS) in MRI, in which Diffusion-weighted imaging (DWI) or apparent diffusion coefficient (ADC) was not involved. The pre-process of center cropping in the original image expanded the proportion of the prostate in the input so that the model paid more attention to the prostate area. Quantitative and robust features combined with artificial intelligence, helped the framework draw a path for decision-making more quickly and accurately. First, to generate a patient-level result, the model's decisions were based not only on a specific single layer but also on the entire "impression" of imaging data at every previous step. This is how the human brain works. Second, the construction of the final decision consisted of both tumor volume and histological aggressiveness information to improve the discriminative power for the final GG (**Supplementary Information II**). For example, when there were two isolated lesions within one case at different layers (minor: 4 + 4; major: 3 + 3), if we choose the slice containing maximum tumor volume as the decision basis, the patient-level decision would be 3 + 3. Otherwise, if we select the layer having the highest score, it would be 4 + 4 (**Figure S3**). Obviously, neither of the aforementioned two answers can be called accurate. However, by adding both tumor volume and histological ranking into the formula, our model successfully optimizes the recipe to mimic patient-level results (3 + 4), which is precisely the way radiologists do it.

Compared with radiomics 48-based machine learning, PCa-GGNet better reflects the characteristics of RP pathology and avoids the restrictions of tumor segmentation. It improved the fitting quality of weakly supervised models and further reduced the dependence of annotated data. The primary principle of our design required the use of high-information entropy input modeling to compensate for the excessive reliance on supervisory information. This system should function well in scenarios that lack domain knowledge, especially for non-prominent tumors such as PCa. The transfer learning method 26 provided a powerful tool to artificial intelligence-based models for expanding advantages of the algorithm to different image types and have been proven in many clinical applications. The prediction model based on mp-MRI for multi-sequence (DWI, ADC, etc.) information joint was hopeful to be further constructed. The framework also has the potential to be extended to other medical image analysis tasks based on different modality images.

On our total dataset, the consistency rate of NB to RP was only 44.9%. Approximately 40.4% of NB cases upgraded to RP, and 14.7% finally downgraded. Patients falling in GG 1 are usually considered for active surveillance (AS) 49. With the increasing percentage of Gleason pattern 4, patients are more likely to be referred to RP and other definitive therapies. Thus, upgrading from GG grade 1 to 2/3 is crucial to the selection between AS and definitive treatment. In our testing cohort, 64.6% of patients in GG 1 experienced upgrading at RP, which indicates that there is a possibility for a group having the same biopsy conditions to face insufficient treatment if they are recommended for AS. In contrast, when considering the implication of extended pelvic lymph node dissection (ePLND), upgrading from GG 2/3 to 4/5 is of great significance. In our testing cohort, over 23% of GG 2 and 53% of GG 3 upgraded to 4/5, meaning that the prognosis of these patients might be compromised, owing to the lack of ePLND. On the contrary, 14% of patients in GG 5 and 35.7% in GG 4 downgraded to GG 3 or below, which suggested that these patients might not benefit from ePLND during radical prostatectomy. Our developed algorithm significantly reduced both upgrading and downgrading for every group. Our model obtained an ACC of approximately 0.85 with internal and external validation. Additionally, due to extended indication of RP in our institutions, we were lucky to have more proportions of lower Gleason patients in our cohorts (15% GG 1), which let our network more adaptive with low-grade GG cases. The critical function of our network was to find out and testify the internal correlations between imaging and pathological appearance. Thus, PCa-GGNet was also potential to be extended to a larger population with multiple objectives such as benign and malignant discrimination, significant and insignificant PCa distinguish tasks, and so on.

To further evaluate the additional benefits in real clinical practice, we stratified all patients in the validation cohort by total PSA, clinical T stage, and GG-NB/GG-Pre and constructed confusion metrics based on the 3-tier protocols of the National Comprehensive Cancer Network (NCCN) (**Supplementary Information III, Figure S4**). When compared with the GG-Pre-based stratifications, 28.4% (23/81) of the medium-risk patients in the GG-NB model would require ePLND for oncological control, and 4.4% (8/180) of the high-risk patients in the GG-NB model might not benefit from ePLND. The distribution differences between groups in the NCCN model were not as high as those in the GG metrics (**Figure 6**), which have the ability to reduce deviations derived from any individual parameter to achieve better performance for the whole system. Nevertheless, by improving the discriminative accuracy GG grading in the current study, we can achieve much better performance of the entire system (approaching the GG-RP model).

Several limitations must be mitigated. First, our current version only involved T2WI-FS data because of image standards and data scales. Future work should include more sequences (e.g., non-fs T2WI, DWI, ADC, and DCE) to provide better multi-tier GG prediction by transfer learning 26 and verify the proposed method on more international public data sets for different clinical applications (e.g., ProstateX dataset 50). Second, multi-center validations at a larger scale of populations and prospective data were considered in the future. Third, the current study only involved systemic biopsy results. Thus, more work needs to be done to explore the discriminative power between our method and the targeted biopsy. Additionally, of all biopsy proved prostate cancer patients, only those who received radical prostatectomy had been enrolled in this study, which brought selection bias neglecting patients without RP surgery. Last, the decision-making process from core-level, specimen-level, to patient-level results is variable. Some doctors tend to adopt the highest core-/specimen-level result for risk evaluation, whereas others tend to select the GG of the index lesion or provide a tertiary clinical decision. Ideally, it might be the best case to report precise percentages of each Gleason pattern for every lesion of all specimens. However, such work is nearly impossible for human efforts in clinical routines. During the deep learning era, further work on the quantitative Gleason pattern 51,52 evaluation at gland- or pixel levels should be explored.

In summary, we proposed a human-like PCa-GGNet framework to predict patients' final GG of RP. According to the multi-center validation, our method demonstrated high reliability of reducing risks of upgrading and downgrading from biopsy to RP pathology. This framework will facilitate clinicians by providing more precise treatment options, and it has the potential for application to other MRI-based tumor research.

## Supplementary Material

Supplementary figures and tables.

## Figures and Tables

**Table 1:** Patient characteristics

	PUTH	PUPH	*P*-value
Number of patients	488	87	
Age, median (IQR)	70 (65)	70 (64)	0.104
Total PSA, median (IQR)	11.5 (7.30)	14.7 (9.09)	0.596
Free PSA, median (IQR)	1.48 (0.87)	1.70 (0.89)	0.855
**Clinical T stage**			0.022
2a-2c	211 (43.2)	51 (58.6)	
3a	175 (35.9)	20 (23.0)	
3b	102 (20.9)	16 (18.4)	
Positive needle, median (IQR)	5 (2)	5 (3)	
**GG-NB, *N* (%)**			0.017
1	123 (25.2)	30 (34.5)	
2	98 (20.1)	23 (26.4)	
3	73 (15.0)	16 (18.4)	
4	99 (20.3)	11 (12.6)	
5	95 (19.5)	7 (8.0)	
**GG-RP, *N* (%)**			0.464
1	68 (13.9)	14 (16.1)	
2	126 (25.8)	26 (29.9)	
3	96 (19.7)	21 (24.1)	
4	75 (15.4)	10 (11.5)	
5	123 (25.2)	16 (18.4)	

Note: PUTH, Peking university third hospital; PUPH, Peking university people's hospital; GG-NB, grade group for pathological assessment of needle biopsy; GG-RP, grade group for pathological assessment of radical prostatectomy; *N*, the number of patients.

## Abbreviations

ACC: five-grade prediction accuracy
CI: confidence interval
CNN: convolutional neural network
DRL: deep reinforcement learning
GS: Gleason score
GG: grade group
GG-NB: GG of needle biopsy
GG-RP: GG of radical prostatectomy
mp MRI: multiparametric magnetic resonance imaging
NB: needle biopsy
PC: primary cohort
RP: radical prostatectomy
T2WI: T2-weighted image
TC: testing cohort
VC: validation cohort

## References

[1] Epstein JI, Feng Z, Trock BJ, Pierorazio PM (2012). Upgrading and downgrading of prostate cancer from biopsy to radical prostatectomy: incidence and predictive factors using the modified Gleason grading system and factoring in tertiary grades. Eur Urol.

[2] Epstein JI, Egevad L, Amin MB, Delahunt B, Srigley JR, Humphrey PA (2016). The 2014 International Society of Urological Pathology (ISUP) Consensus Conference on Gleason Grading of Prostatic Carcinoma: Definition of Grading Patterns and Proposal for a New Grading System. Am J Surg Pathol.

[3] Loeb S, Folkvaljon Y, Robinson D, Lissbrant IF, Egevad L, Stattin P (2016). Evaluation of the 2015 Gleason Grade Groups in a Nationwide Population-based Cohort. Eur Urol.

[4] Epstein JI, Zelefsky MJ, Sjoberg DD, Nelson JB, Egevad L, Magi-Galluzzi C (2016). A Contemporary Prostate Cancer Grading System: A Validated Alternative to the Gleason Score. Eur Urol.

[5] He J, Albertsen PC, Moore D, Rotter D, Demissie K, Lu-Yao G (2017). Validation of a Contemporary Five-tiered Gleason Grade Grouping Using Population-based Data. Eur Urol.

[6] Evans SM, Patabendi BV, Kronborg C, Earnest A, Millar J, Clouston D (2016). Gleason group concordance between biopsy and radical prostatectomy specimens: A cohort study from Prostate Cancer Outcome Registry-Victoria. Prostate Int.

[7] Muntener M, Epstein JI, Hernandez DJ, Gonzalgo ML, Mangold L, Humphreys E (2008). Prognostic significance of Gleason score discrepancies between needle biopsy and radical prostatectomy. Eur Urol.

[8] Rajinikanth A, Manoharan M, Soloway CT, Civantos FJ, Soloway MS (2008). Trends in Gleason score: concordance between biopsy and prostatectomy over 15 years. Urology.

[9] Gandaglia G, Ploussard G, Valerio M, Mattei A, Fiori C, Roumiguie M (2020). The Key Combined Value of Multiparametric Magnetic Resonance Imaging, and Magnetic Resonance Imaging-targeted and Concomitant Systematic Biopsies for the Prediction of Adverse Pathological Features in Prostate Cancer Patients Undergoing Radical Prostatectomy. Eur Urol.

[10] Vos EK, Litjens GJ, Kobus T, Hambrock T, Hulsbergen-van DKC, Barentsz JO (2013). Assessment of prostate cancer aggressiveness using dynamic contrast-enhanced magnetic resonance imaging at 3 T. Eur Urol.

[11] Mehralivand S, Shih JH, Rais-Bahrami S, Oto A, Bednarova S, Nix JW (2018). A Magnetic Resonance Imaging-Based Prediction Model for Prostate Biopsy Risk Stratification. Jama Oncol.

[12] Wang Q, Li H, Yan X, Wu CJ, Liu XS, Shi HB (2015). Histogram analysis of diffusion kurtosis magnetic resonance imaging in differentiation of pathologic Gleason grade of prostate cancer. Urol Oncol.

[13] Donati OF, Afaq A, Vargas HA, Mazaheri Y, Zheng J, Moskowitz CS (2014). Prostate MRI: evaluating tumor volume and apparent diffusion coefficient as surrogate biomarkers for predicting tumor Gleason score. Clin Cancer Res.

[14] Schelb P, Kohl S, Radtke JP, Wiesenfarth M, Kickingereder P, Bickelhaupt S (2019). Classification of Cancer at Prostate MRI: Deep Learning versus Clinical PI-RADS Assessment. Radiology.

[15] Liu Z, Wang S, Dong D, Wei J, Fang C, Zhou X (2019). The Applications of Radiomics in Precision Diagnosis and Treatment of Oncology: Opportunities and Challenges. Theranostics.

[16] Chaddad A, Kucharczyk MJ, Niazi T (2018). Multimodal Radiomic Features for the Predicting Gleason Score of Prostate Cancer. Cancers.

[17] Nketiah G, Elschot M, Kim E, Teruel JR, Scheenen TW, Bathen TF (2017). T2-weighted MRI-derived textural features reflect prostate cancer aggressiveness: preliminary results. Eur Radiol.

[18] Jia P, Xue H, Liu S, Wang H, Yang L, Hesketh T (2019). Opportunities and challenges of using big data for global health. Sci Bull.

[19] Campanella G, Hanna MG, Geneslaw L, Miraflor A, Werneck KSV, Busam KJ (2019). Clinical-grade computational pathology using weakly supervised deep learning on whole slide images. Nat Med.

[20] Lucas M, Jansen I, Savci-Heijink CD, Meijer SL, de Boer OJ, van Leeuwen TG (2019). Deep learning for automatic Gleason pattern classification for grade group determination of prostate biopsies. Virchows Arch.

[21] Cao R, Mohammadian BA, Afshari MS, Shakeri S, Zhong X, Enzmann D (2019). Joint Prostate Cancer Detection and Gleason Score Prediction in mp-MRI via FocalNet. IEEE Trans Med Imaging.

[22] Mnih V, Kavukcuoglu K, Silver D, Rusu AA, Veness J, Bellemare MG (2015). Human-level control through deep reinforcement learning. Nature.

[23] Mahmud M, Kaiser MS, Hussain A, Vassanelli S (2018). Applications of Deep Learning and Reinforcement Learning to Biological Data. Ieee T Neur Net Lear.

[24] Higa S, Iwashita Y, Otsu K, Ono M, Lamarre O, Didier A (2019). Vision-Based Estimation of Driving Energy for Planetary Rovers Using Deep Learning and Terramechanics. IEEE ROBOTICS AND AUTOMATION LETTERS.

[25] Russakovsky O, Deng J, Su H, Krause J, Satheesh S, Ma S (2015). ImageNet Large Scale Visual Recognition Challenge. Int J Comput Vision.

[26] Shin HC, Roth HR, Gao M, Lu L, Xu Z, Nogues I (2016). Deep Convolutional Neural Networks for Computer-Aided Detection: CNN Architectures, Dataset Characteristics and Transfer Learning. IEEE Trans Med Imaging.

[27] Cohen MS, Hanley RS, Kurteva T, Ruthazer R, Silverman ML, Sorcini A (2008). Comparing the Gleason Prostate Biopsy and Gleason Prostatectomy Grading System: The Lahey Clinic Medical Center Experience and an International Meta-Analysis. Eur Urol.

[28] Corcoran NM, Hong MK, Casey RG, Hurtado-Coll A, Peters J, Harewood L (2011). Upgrade in Gleason score between prostate biopsies and pathology following radical prostatectomy significantly impacts upon the risk of biochemical recurrence. Bju Int.

[29] Boorjian SA, Karnes RJ, Crispen PL, Rangel LJ, Bergstralh EJ, Sebo TJ (2009). The impact of discordance between biopsy and pathological Gleason scores on survival after radical prostatectomy. J Urol.

[30] Bakavicius A, Drevinskaite M, Daniunaite K, Barisiene M, Jarmalaite S, Jankevicius F (2020). The Impact of Prostate Cancer Upgrading and Upstaging on Biochemical Recurrence and Cancer-Specific Survival. Medicina.

[31] Chun FK, Steuber T, Erbersdobler A, Currlin E, Walz J, Schlomm T (2006). Development and internal validation of a nomogram predicting the probability of prostate cancer Gleason sum upgrading between biopsy and radical prostatectomy pathology. Eur Urol.

[32] Corcoran NM, Hovens CM, Hong MK, Pedersen J, Casey RG, Connolly S (2012). Underestimation of Gleason score at prostate biopsy reflects sampling error in lower volume tumours. Bju Int.

[33] Thomas C, Pfirrmann K, Pieles F, Bogumil A, Gillitzer R, Wiesner C (2012). Predictors for clinically relevant Gleason score upgrade in patients undergoing radical prostatectomy. Bju Int.

[34] Kasivisvanathan V, Emberton M, Moore CM (2018). MRI-Targeted Biopsy for Prostate-Cancer Diagnosis. N Engl J Med.

[35] Goel S, Shoag JE, Gross MD, Al HAAB, Robinson B, Khani F (2020). Concordance Between Biopsy and Radical Prostatectomy Pathology in the Era of Targeted Biopsy: A Systematic Review and Meta-analysis. Eur Urol Oncol.

[36] Quentin M, Blondin D, Arsov C, Schimmoller L, Hiester A, Godehardt E (2014). Prospective evaluation of magnetic resonance imaging guided in-bore prostate biopsy versus systematic transrectal ultrasound guided prostate biopsy in biopsy naive men with elevated prostate specific antigen. J Urol.

[37] Valerio M, Donaldson I, Emberton M, Ehdaie B, Hadaschik BA, Marks LS (2015). Detection of Clinically Significant Prostate Cancer Using Magnetic Resonance Imaging-Ultrasound Fusion Targeted Biopsy: A Systematic Review. Eur Urol.

[38] Elkhoury FF, Felker ER, Kwan L, Sisk AE, Delfin M, Natarajan S (2019). Comparison of Targeted vs Systematic Prostate Biopsy in Men Who Are Biopsy Naive: The Prospective Assessment of Image Registration in the Diagnosis of Prostate Cancer (PAIREDCAP) Study. Jama Surg.

[39] Arvaniti E, Fricker KS, Moret M, Rupp N, Hermanns T, Fankhauser C (2018). Automated Gleason grading of prostate cancer tissue microarrays via deep learning. Sci Rep.

[40] Lucas M, Jansen I, Savci-Heijink CD, Meijer SL, de Boer OJ, van Leeuwen TG (2019). Deep learning for automatic Gleason pattern classification for grade group determination of prostate biopsies. Virchows Arch.

[41] Nguyen TH, Sridharan S, Macias V, Kajdacsy-Balla A, Melamed J, Do MN (2017). Automatic Gleason grading of prostate cancer using quantitative phase imaging and machine learning. J Biomed Opt.

[42] Antonelli M, Johnston EW, Dikaios N, Cheung KK, Sidhu HS, Appayya MB (2019). Machine learning classifiers can predict Gleason pattern 4 prostate cancer with greater accuracy than experienced radiologists. Eur Radiol.

[43] Abraham B, Nair MS (2018). Computer-aided classification of prostate cancer grade groups from MRI images using texture features and stacked sparse autoencoder. Comput Med Imaging Graph.

[44] Citak-Er F, Vural M, Acar O, Esen T, Onay A, Ozturk-Isik E (2014). Final Gleason score prediction using discriminant analysis and support vector machine based on preoperative multiparametric MR imaging of prostate cancer at 3T. Biomed Res Int.

[45] Fehr D, Veeraraghavan H, Wibmer A, Gondo T, Matsumoto K, Vargas HA (2015). Automatic classification of prostate cancer Gleason scores from multiparametric magnetic resonance images. Proc Natl Acad Sci U S A.

[46] Litjens G, Kooi T, Bejnordi BE, Setio A, Ciompi F, Ghafoorian M (2017). A survey on deep learning in medical image analysis. Med Image Anal.

[47] Petter EA, Gershman SJ, Meck WH (2018). Integrating Models of Interval Timing and Reinforcement Learning. Trends Cogn Sci.

[48] Aerts HJ, Velazquez ER, Leijenaar RT, Parmar C, Grossmann P, Carvalho S (2014). Decoding tumour phenotype by non-invasive imaging using a quantitative radiomics approach. Nat Commun.

[49] Chun FK, Haese A, Ahyai SA, Walz J, Suardi N, Capitanio U (2008). Critical assessment of tools to predict clinically insignificant prostate cancer at radical prostatectomy in contemporary men. Cancer-Am Cancer Soc.

[50] Armato SGR, Huisman H, Drukker K, Hadjiiski L, Kirby JS, Petrick N (2018). PROSTATEx Challenges for computerized classification of prostate lesions from multiparametric magnetic resonance images. Journal of medical imaging (Bellingham, Wash.).

[51] Sauter G, Steurer S, Clauditz TS, Krech T, Wittmer C, Lutz F (2016). Clinical utility of quantitative Gleason grading in prostate biopsies and prostatectomy specimens. Eur Urol.

[52] Egevad L, Ahmad AS, Algaba F, Berney DM, Boccon Gibod L, Compérat E (2013). Standardization of Gleason grading among 337 European pathologists. Histopathology.

