# Supplementary material for: Radiologist-like artificial intelligence for grade group prediction of radical prostatectomy for reducing upgrading and downgrading from biopsy
Source: Theranostics. 2020 Sep 2;10(22):10200–12. doi: 10.7150/thno.48706 (PMC7481433; doi:10.7150/thno.48706)
Supplement: Supplementary file 1 — Supplementary figures and tables. [file thnov10p10200s1.pdf]

Supplementary Information

I. **Details of annotations for T2-weighted image by whole mount prostatectomy section**

II. **Visualization of interpretable predictions of the method**

III. **Assessment of potential clinical treatment benefits**

I. Details of annotations for T2-weighted image by whole mount prostatectomy section

As a multifocal disease, radio-pathological correlation is the gold standard for imaging analysis of prostate cancer. In our clinical practice, one prostate gland was resected transversely into 6 to 8 whole-mount sections, subsequently, those whole-mount sections were resected into 4 to 6 small pieces to fit the paraffin fixation cage. Our pathologists scraped those pieces back into whole-mount slides. Then, we delineated lesions on patched whole-mount slides. Only lesions responsible for final GG assessment are delineated. Very small satellite lesions or lesions contribute little to the final diagnosis are ignored. Finally, our pathologist and radiologist together recognized the correlated lesions on MR images, by using the knowledge of shape, texture, location of both the prostate and the tumors, which is knowing as cognitive registration. Of note, during the MR scan, the axial plane was obtained straight to the patient, while in pathological setting, the prostate was axially sectioned straight to the prostate itself (which is oblique to the patient's body). Thus, there is an angle of 5 to 10 degrees between the MR axial plane and pathological axial plane, which makes perfect registration almost impossible. We aim to make the best effort to match those lesions between MR images and pathological sections (See workflow below). More data and examples of annotations match between prostatectomy sections and T2WI-FS was introduced in our Github (https://github.com/StandWisdom/PCa-GGNet/tree/master/data/samples).

II. Visualization of interpretable predictions of the method

PCa-GGNet imitated the reading habits of human radiologists to identify the key slice (i.e., attentional slice) that best characterized the case-level GG-Pre through model reasoning ability. To illustrate this process, five typical cases were illustrated for visualization and semantic interpretation of the modeling process (**Figure S2**). These five cases involved five patients with different GG-Pre, and demonstrated the model's resilience under circumstances of different initial slices, single or multifocal lesions, different termination-action status, and different decision paths. For example (**Figure S2A**), the patient had multiple foci rated in grade 1. The tumor foci appeared in slice 8-9 (smaller) and 6-4 (larger). The initial attentional slice was randomly selected (slice 8). PCa-GGNet moved the model's attention to the level with the larger lesion and higher predictive probability through three actions, which skipped the slices without decision-making tumoral information. After obtaining the action status of "stay in the place," the decision basis was set at the sixth layer of the patient's T2WI image. The GG-Pre generated from this layer was used as the "case-level" result. Additionally, we listed a case where the initial slice without any tumoral information (**Figure S2E**). In this case, by constantly updating the attention level, the tumor-related slice was finally identified with a higher probability of prediction. In most cases, the attentional slice searching strategy preferred slices with both larger tumoral areas and higher prediction probabilities at the same time. For the case in **Figure S2C**, our proposed method also paid attention not only to the radiological characteristics but also to peritumoral surroundings to draw a more accurate prediction at case-level scale.

III. Assessment of potential clinical treatment benefits

Based on GG-NB, GG-Pre, and the GG-RP, we divided patients into high-, medium-, and low-risk groups for staging according to the NCCN guidelines. The stratification scheme combined the PSA, clinical T stage, GG grade, and other clinicopathological parameters of patients. The control group was recommended by standard NCCN 3-tier categories based on GG-NB. Next, potential treatment changes were compared between GG-NB, GG-Pre, and GG-RP **(Figure S4)**. Compared with GG-NB based NCCN stratifications, 75% of patients in the low-risk group (P=0.144) and 44% of patients in medium-risk (P<0.001) were underestimated. 4% of patients in the high-risk group had a risk of overtreatment(P=0.038). Nealy 29% (23/81) of medium-risk patients in the GG-NB based model would require ePLND for oncological control, while 4.4% (8/180) high-risk patients in GG-NB model might not benefit from ePLND.

Note: The acceptance of Active Surveillance among Chinese patients is not as popular as in Europe and the USA. Chinese patients tend to prefer surgical operations rather than radiotherapy or AS despite the limited potential benefits of GG 1 characteristics. This is part of the reason why such amount of people in GG 1 received RP instead of AS or ERBT. Restrained by a retrospective study manner, we were incapable to alter the past. However, such facts supported our study with much more low-risk data than it should be.

Supplementary Tables

I. Table S1
II. Table S2
III. Table S3
IV. Table S4
V. Table S5
VI. Table S6
VII. Table S7

Table S1. MRI Parameters

	PUTH		PUPH
	Scanner 1	Scanner 2	Scanner 3
**Manufacturer**	Siemens Healthcare , Erlangen, Germany	General Electric, Milwaukee, USA	General Electric, Milwaukee, USA
**Model**	3T Trio Tim	3T Discovery MR750	3T Discovery MR750
**Coils**	None	None	None
**T2 weighted imaging**			
Repetition Time/Echo Time	3600/80	6083/99	4160/131
Reconstruction Voxel Spacing (mm3)	0.625*0.625*4.8	0.469*0.469*4.5	0.352*0.352*4
Acquisition time (min)	2.03	2.73	2.59
**Diffusion-weighted imaging**			
Repetition Time/Echo Time	3600/80	4000/76	4200/75
Acquisition voxel size (mm3)	2.187*2.187*4	1.875*1.875*4	1.875*1.875*4
B-values (s/mm2)	0, 200, 400, 800, 1000	0, 50, 100, 200, 400, 800, 1500, 2000	0, 50, 100, 200, 400, 800, 1500
Acquisition time (min)	3.01	4.30	4.21

Note: PUTH, Peking University Third Hospital; PUPH, Peking University People Hospital.

Table S2. GG of radical prostatectomy distribution of the data sets.

N (%)	1	2	3	4	5
PC	56 (18.1)	84 (27.1)	56 (18.1)	42 (13.5)	72 (23.2)
VC	5 (16.7)	12 (40)	4 (13.3)	4 (13.3)	5 (16.7)
TC1	13 (7.3)	43 (24.2)	39 (21.9)	30 (16.9)	53 (29.8)
TC2	14 (16.1)	26 (29.9)	21 (24.1)	10 (11.5)	16 (18.4)

Note: GG, grade group; PC, primary cohort; VC, validation cohort; TC1, testing cohort1 (internal verification); TC2, testing cohort2 (external center verification). *N*, the number of samples. (%), the proportion of samples in the data set.

Table S3. Assessment of consistency by accuracy (ACC)

ACC (95% CI)	PC	VC	TC1	TC2
GG-NB	0.478 (0.417-0.54)	0.519 (0.337-0.701)	0.500 (0.427-0.572)	0.437 (0.335-0.539)
PCa-GGNet	0.847 (0.826-0.867)	0.83 (0.762-0.898)	0.781 (0.751-0.811)	0.815 (0.773-0.857)

Note: GG-NB, GG for pathological assessment of needle biopsy. PC, primary cohort; VC, validation cohort; TC1, testing cohort1 (internal verification); TC2, testing cohort2 (external center verification).

Table S4. Five-category accuracy of Generator-net for predicting GG-RP in slice-level

ACC (95%CI)	Grade		Overall		Grade | Overall	
	PC	VC	PC	VC	PC	VC
Densenet121	0.673 (0.652-0.694)	0.603 (0.534-0.671)	0.757 (0.747-0.767)	0.70 (0.669-0.732)	0.346 (0.325-0.368)	0.335 (0.264-0.405)
pnasnet5large	0.73 (0.711-0.75)	0.615 (0.545-0.686)	0.838 (0.83-0.847)	0.803 (0.777-0.829)	0.54 (0.517-0.562)	0.523 (0.451-0.594)
resnext101	0.636 (0.615-0.657)	0.525 (0.455-0.596)	0.784 (0.773-0.794)	0.708 (0.675-0.74)	0.461 (0.438-0.483)	0.353 (0.284-0.422)
inceptionresnetv2	0.642 (0.62-0.664)	0.434 (0.361-0.507)	0.824 (0.814-0.833)	0.766 (0.736-0.797)	0.446 (0.423-0.468)	0.336 (0.27-0.403)

Note: GG-RP, grade group of radical prostatectomy. Overall, the overall accuracy of six-category classifier for distinguishing five-grade and slice without tumor; Grade | Overall, The prediction accuracy of the five-grade in the prediction results of the six-category classifier. PC, primary cohort; VC, validation cohort.

Table S5. Assessemt of PNASNet-5large at each grade of GG-RP

	Metrics	PC	VC
Grade 1	Precision	0.61	0.56
	Recall	0.82	0.84
	F1score	0.70	0.67
Grade 2	Precision	0.85	0.91
	Recall	0.59	0.51
	F1score	0.70	0.65
Grade 3	Precision	0.64	0.44
	Recall	0.79	0.67
	F1score	0.71	0.53
Grade 4	Precision	0.74	0.57
	Recall	0.65	0.44
	F1score	0.69	0.50
Grade 5	Precision	0.82	0.59
	Recall	0.80	0.68
	F1score	0.81	0.63

Note: GG-RP, grade group of radical prostatectomy; PC, primary cohort; VC, validation cohort.

Table S6. Evaluation of action-net for attention slices

	PC	VC
ACC (95%CI)	0.861 (0.849-0.873)	0.797 (0.754-0.841)
SEN (95%CI)	1.0 (1.0-1.0)	1.0 (1.0-1.0)
SPE (95%CI)	0.86 (0.848-0.872)	0.797 (0.754-0.841)
ACC_{GG} (95%CI)	0.86 (0.846-0.874)	0.832 (0.784-0.88)

Note: SEN, sensitivity; SPE, specificity; ACC_{GG}, ACC of GG for selected attention slices; PC, primary cohort; VC, validation cohort.

Table S7. Performance of PCa-GGNet at each grade of GG-RP

	Metrics	PC	VC	TC1	TC2
1	Precision	0.976(0.953-0.999)	0.826(0.653-0.999)	0.797(0.663-0.931)	1.0(1.0-1.0)
	Recall	0.803(0.75-0.857)	0.995(0.926-1.064)	0.616(0.473-0.759)	0.787(0.678-0.896)
	F1-score	0.88(0.846-0.915)	0.893(0.767-1.018)	0.684(0.566-0.803)	0.876(0.805-0.947)
2	Precision	0.799(0.755-0.844)	0.765(0.647-0.884)	0.638(0.572-0.703)	0.709(0.626-0.791)
	Recall	0.875(0.837-0.913)	0.831(0.721-0.942)	0.815(0.756-0.874)	0.849(0.781-0.918)
	F1-score	0.835(0.802-0.868)	0.79(0.697-0.883)	0.713(0.66-0.766)	0.769(0.707-0.832)
3	Precision	0.893(0.846-0.939)	0.881(0.567-1.196)	0.929(0.879-0.979)	0.854(0.774-0.934)
	Recall	0.788(0.731-0.845)	0.509(0.227-0.79)	0.639(0.564-0.714)	0.806(0.72-0.892)
	F1-score	0.835(0.794-0.876)	0.62(0.341-0.899)	0.755(0.696-0.813)	0.826(0.76-0.892)
4	Precision	0.825(0.76-0.891)	0.796(0.603-0.989)	0.812(0.736-0.888)	0.992(0.905-1.079)
	Recall	0.738(0.668-0.808)	0.989(0.894-1.083)	0.731(0.652-0.81)	0.492(0.325-0.658)
	F1-score	0.777(0.723-0.83)	0.868(0.725-1.012)	0.766(0.704-0.828)	0.641(0.482-0.8)
5	Precision	0.81(0.768-0.853)	0.988(0.893-1.082)	0.832(0.784-0.88)	0.797(0.707-0.887)
	Recall	0.954(0.929-0.98)	0.81(0.617-1.002)	0.925(0.888-0.962)	1.0(1.0-1.0)
	F1-score	0.876(0.848-0.904)	0.877(0.732-1.022)	0.875(0.842-0.907)	0.884(0.826-0.942)

Note: GG-RP, grade group of radical prostatectomy; PC, primary cohort; VC, validation cohort; TC1, testing cohort 1, TC2, testing cohort 2.

Supplementary Figures

VIII. Figure S1
IX. Figure S2
X. Figure S3
XI. Figure S4

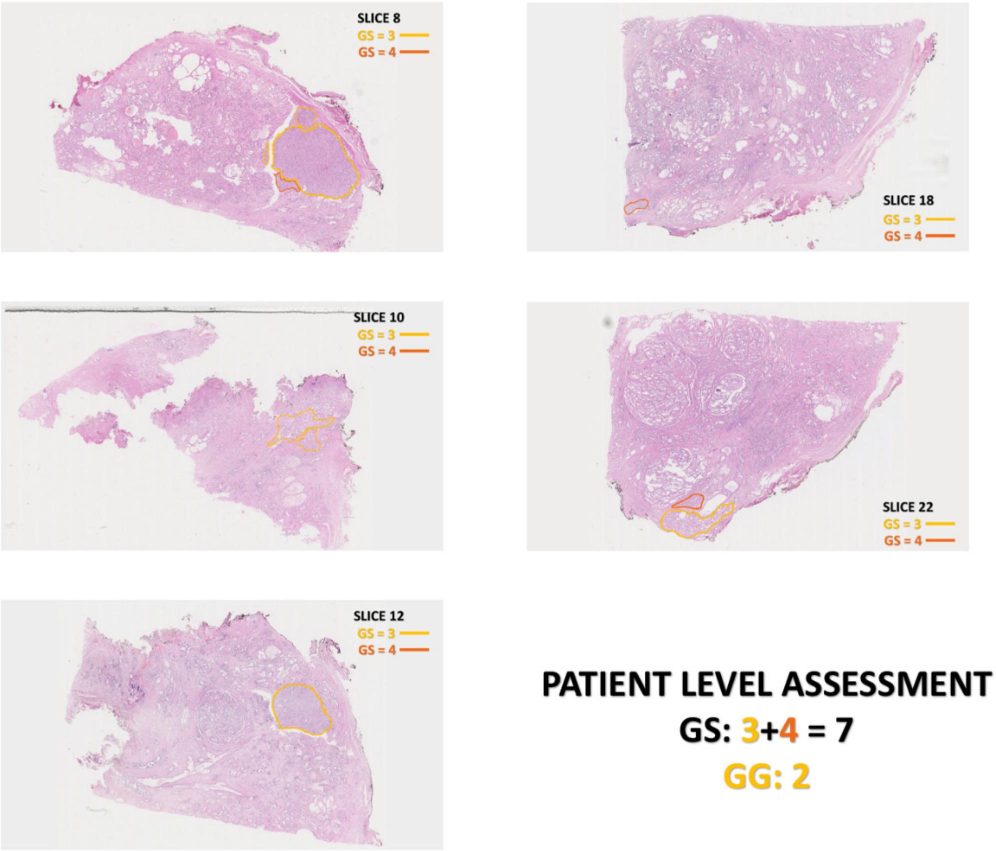

Figure S1. Pathological evaluation of the prostatectomy section.

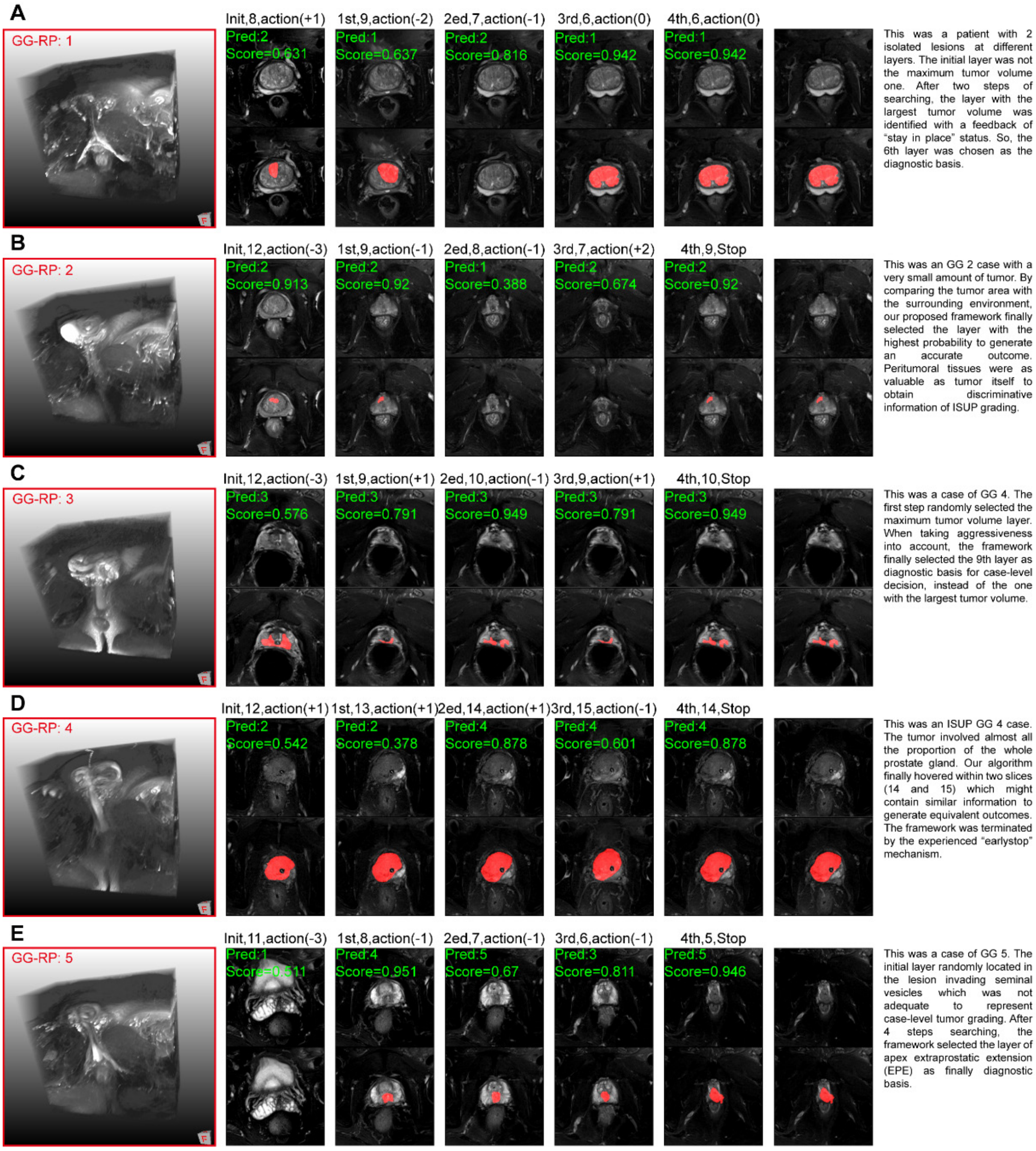

Figure S2. Example of five patients with selected slice predicted by PCa-GGNet, for each grade (1-5) under different conditions, respectively. The first column shows the 3D preview of T2WI. The first row of second to sixth columns showed the selected images as inputs and their predicted probability. The second row showed real tumor location according to postoperative pathology. The title of each column indicated the current status, slice id, and status of the action. Green words were the prediction result of the current slice. To observe the robustness of the model, we deliberately selected two cases in which the initial layer was not the middle layer for display (A, E).

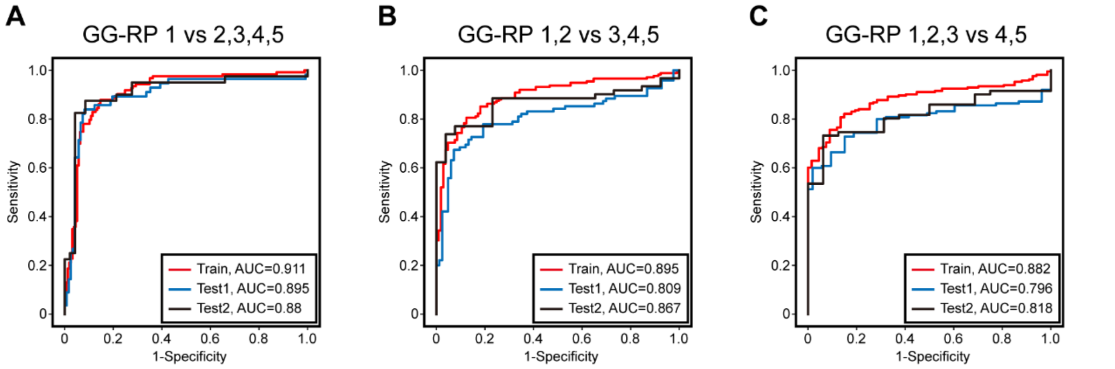

Figure S3. ROCs of subgroups of GG-RP. **(A)** low-grade, grade 1 vs 2,3,4,5. **(B)** Medium-grade, grade 1,2 vs 3,4,5. **(C)** High-grade, grade 1,2,3 vs 4,5.

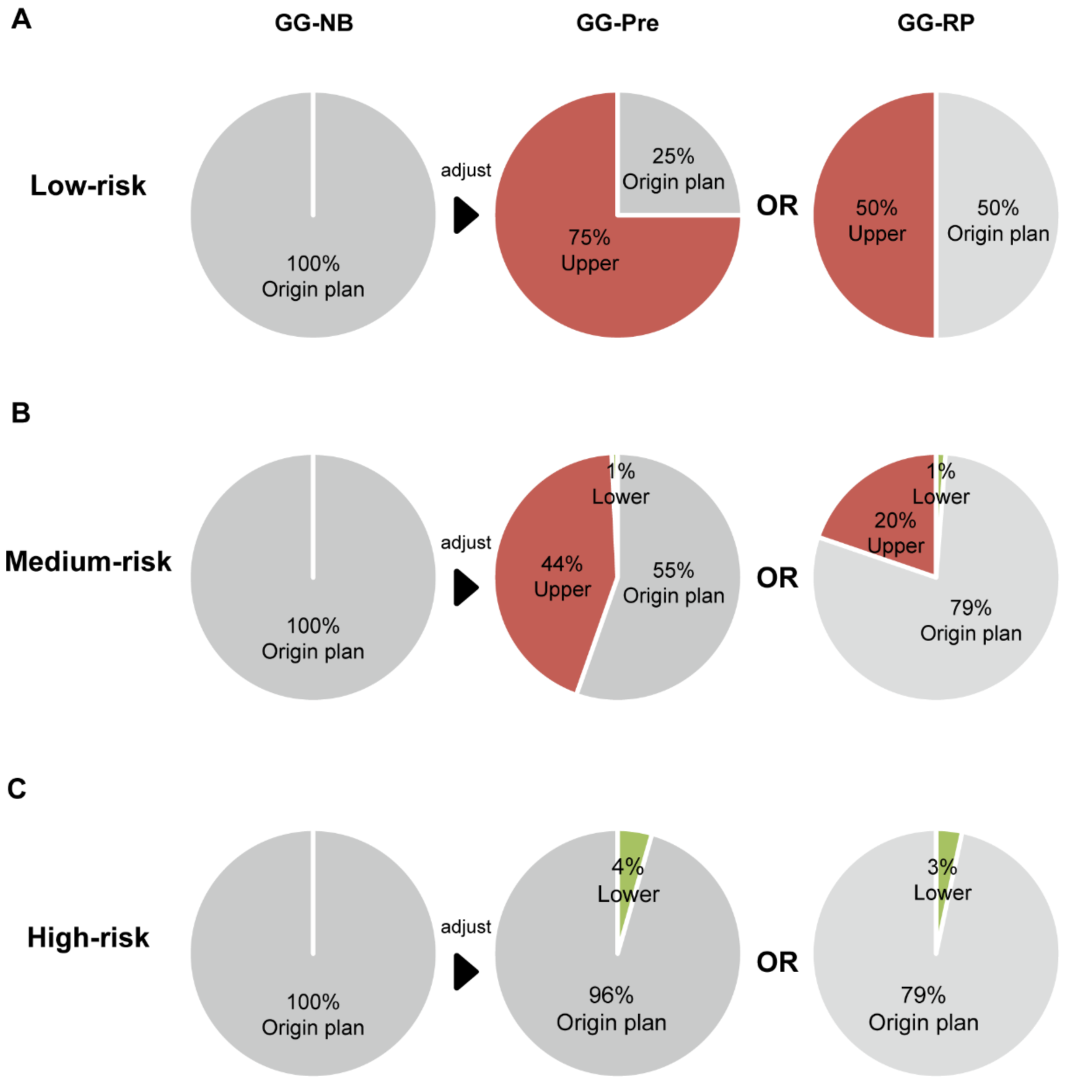

Figure S4. Potential clinical benefits of GG-Pre from our method and GG-NB from the biopsy. **(A)** Low-risk of the NCCN guideline. According to the GG-Pre based model, 75% of low-risk patients upgraded to the medium-risk group, and definitive therapies are more appropriate rather than active surveillance. While the true upgrading rate was 50% according to the GG-RP model. **(B)** Medium-risk. According to the GG-Pre model, 44% of cases upgraded to the high-risk group, in which extended pelvic lymph node dissection might be needed. While the true upgrading rate was 20% by the GG-RP model. **(C)** High-risk. Marginal benefits were demonstrated in NCCN high-risk group.

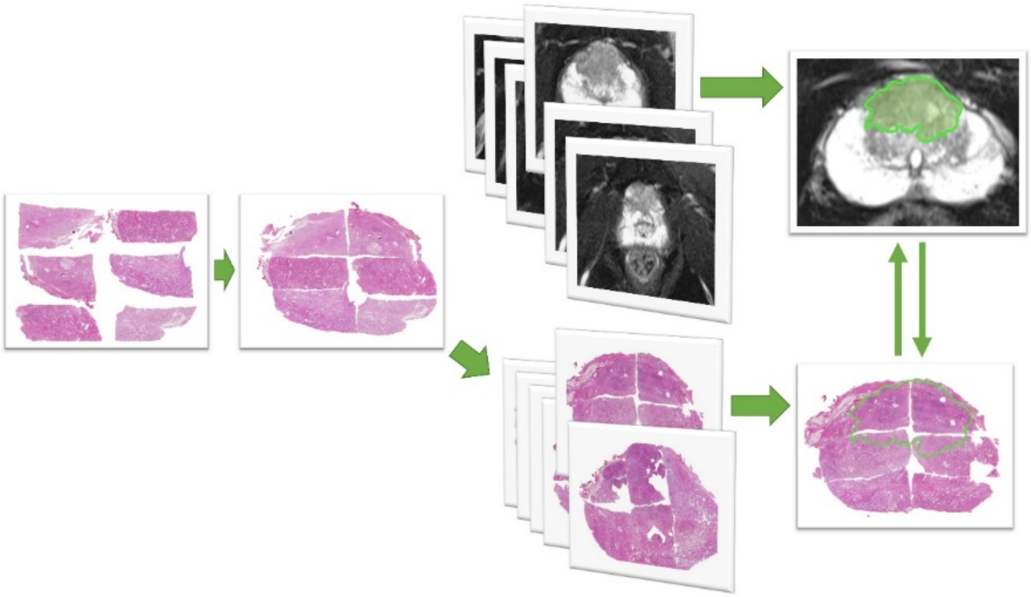

Figure S5. The workflow of annotations.

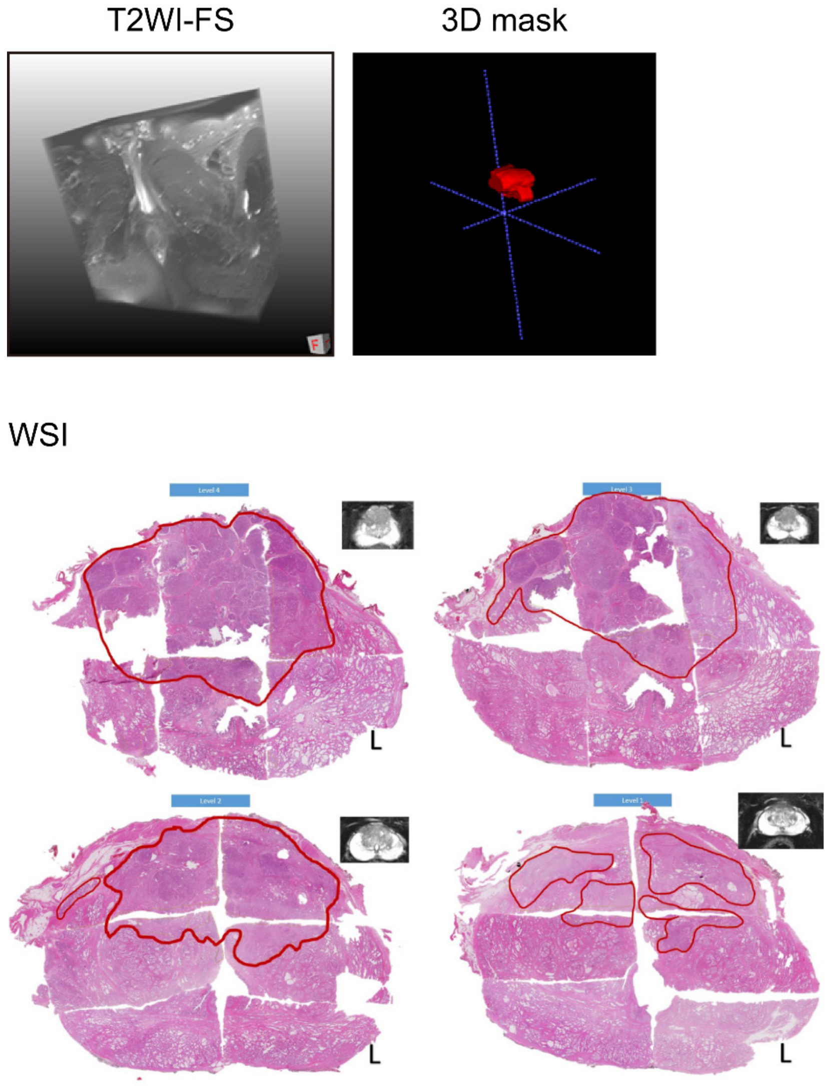

Figure S6. An example of annotation match between prostatectomy sections and T2WI-FS
